# Aqua­(3-fluoro­benzoato-κ*O*)(3-fluoro­benzoato-κ^2^ *O*,*O*′)(1,10-phenanthroline-κ^2^ *N*,*N*′)cobalt(II)

**DOI:** 10.1107/S1600536811051804

**Published:** 2011-12-07

**Authors:** Xiao-Hui Wang, Li-Mei Sun

**Affiliations:** a College of Chemistry and Chemical Engineering, Inner Mongolia University for Nationalities, 028042 Tongliao, Inner Mongolia, People’s Republic of China

## Abstract

In the title compound, [Co(C_7_H_4_FO_2_)_2_(C_12_H_8_N_2_)(H_2_O)], the Co^II^ ion is coordinated by two O atoms from one 3-fluoro­benzoate (fb) ligand and one O atom from another fb ligand, two N atoms from the 1,10-phenanthroline ligand and a water mol­ecule in a distorted octa­hedral geometry. An intra­molecular O—H⋯O hydrogen bond occurs. Inter­molecular O—H⋯O hydrogen bonds link pairs of mol­ecules into centrosymmetric dimers. Weak inter­molecular C—H⋯O and C—H⋯F hydrogen bonds and π–π inter­actions between the aromatic rings [shortest centroid–centroid distance = 3.4962 (2) Å] further stabilize the crystal packing.

## Related literature

For the crystal structures of related metal complexes with 3-fluoro­benzoic acid, see: Sevryugina *et al.* (2007 ▶); Motokawa *et al.* (2008 ▶); Wein *et al.* (2009 ▶); Yin (2011 ▶); Miyasaka *et al.* (2011 ▶).
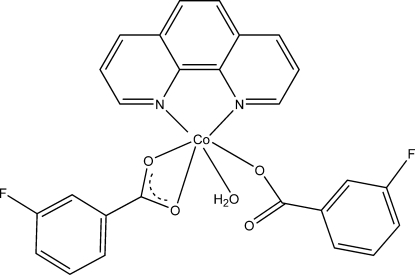


## Experimental

### 

#### Crystal data

[Co(C_7_H_4_FO_2_)_2_(C_12_H_8_N_2_)(H_2_O)]
*M* *_r_* = 535.35
Triclinic, 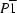

*a* = 8.6517 (7) Å
*b* = 12.1233 (10) Å
*c* = 12.6752 (10) Å
α = 64.045 (1)°
β = 88.879 (1)°
γ = 72.892 (1)°
*V* = 1133.22 (16) Å^3^
*Z* = 2
Mo *K*α radiation
μ = 0.82 mm^−1^
*T* = 298 K
0.30 × 0.20 × 0.12 mm

#### Data collection

Bruker SMART APEXII CCD area-detector diffractometer
Absorption correction: multi-scan (*SADABS*; Bruker, 2005 ▶) *T*_min_ = 0.792, *T*_max_ = 0.908
4724 measured reflections
3980 independent reflections
3119 reflections with *I* > 2σ(*I*)
*R*_int_ = 0.032

#### Refinement

*R*[*F* ^2^ > 2σ(*F* ^2^)] = 0.038
*wR*(*F* ^2^) = 0.105
*S* = 1.03
3980 reflections
333 parameters
H atoms treated by a mixture of independent and constrained refinement
Δρ_max_ = 0.73 e Å^−3^
Δρ_min_ = −0.39 e Å^−3^

### 

Data collection: *APEX2* (Bruker, 2005 ▶); cell refinement: *SAINT* (Bruker, 2005 ▶); data reduction: *SAINT*; program(s) used to solve structure: *SHELXTL* (Sheldrick, 2008 ▶); program(s) used to refine structure: *SHELXTL*; molecular graphics: *SHELXTL*; software used to prepare material for publication: *SHELXTL*.

## Supplementary Material

Crystal structure: contains datablock(s) global, I. DOI: 10.1107/S1600536811051804/cv5209sup1.cif

Structure factors: contains datablock(s) I. DOI: 10.1107/S1600536811051804/cv5209Isup2.hkl

Additional supplementary materials:  crystallographic information; 3D view; checkCIF report

## Figures and Tables

**Table 1:** Hydrogen-bond geometry (Å, °)

*D*—H⋯*A*	*D*—H	H⋯*A*	*D*⋯*A*	*D*—H⋯*A*
O5—H1⋯O4	0.85 (1)	1.76 (1)	2.584 (3)	165 (3)
O5—H2⋯O1^i^	0.85 (1)	1.89 (1)	2.734 (3)	176 (3)
C22—H22⋯O5^ii^	0.93	2.52	3.332 (4)	147
C5—H5*A*⋯F2^iii^	0.93	2.55	3.303 (4)	138

Symmetry codes: (i) 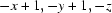; (ii) 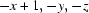; (iii) 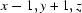.

**Fig. 1.**
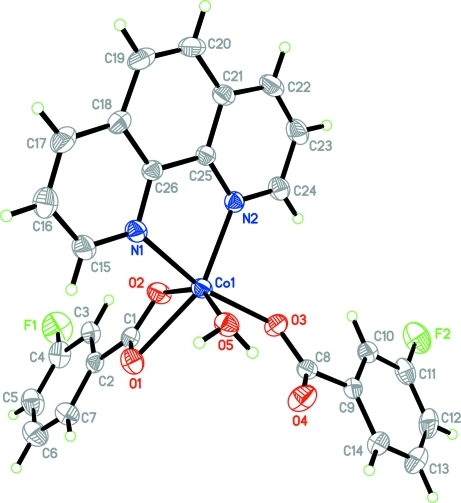
The molecular structure of (I), with displacement ellipsoids drawn at the 30% probability level.

**Fig. 2.**
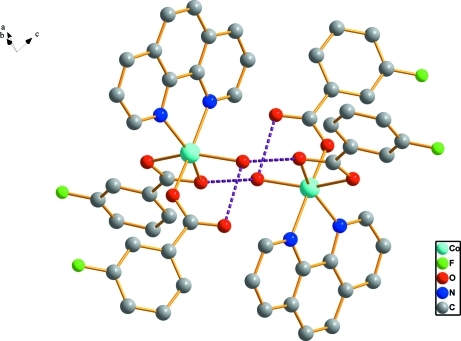
The dimer structure of (I). All H atoms have been omitted for clarity. The dashed lines indicate the O—H···O hydrogen bonds.

## supplementary crystallographic information

### Comment

In recent years, the design and synthesis of metal complexes based on 3-fluorobenzoic acid have attracted much attention (Sevryugina *et al.*, 2007; Motokawa *et al.*, 2008; Wein *et al.*, 2009; Yin, 2011; Miyasaka *et al.*, 2011). We report herein the crystal structure of the title compound (I) (Fig. 1) based on 3-fluorobenzoic acid and 1,10-phenanthroline.

In the crystal structure, the adjacent mononuclear units are linked into a centrosymmetric dimer structure *via* O—H···O hydrogen bonds (Fig. 2). Furthermore, intramolecular weak intermolecular C—H···O(F) hydrogen bonds (Table 1) and π—π interactions between the aromatic rings [shortest centroid–centroid distance = 3.4962 (2) Å] stabilize the crystal structure.

### Experimental

A mixture of Co(CH_3_COO)_2_^.^4H_2_O (0.1 mmol), 3-fluorobenzoic acid (0.2 mmol), Et_3_N (0.1 ml), EtOH (10 ml) and H_2_O (5 ml) was sealed in a 25 ml Teflon-lined stainless-steel reactor, heated to 393 K for 72 h, and then slowly cooled to room temperature. Purple block crystals suitable for X-ray diffraction analysis were collected by filtration.

### Refinement

H atoms attached to C atoms were placed in calculated positions (C—H = 0.93 Å) and refined as riding atoms, with *U*_iso_(H) = 1.2 *U*_eq_(C). The water H atoms were located in a difference map and refined with O—H bond length restrained to 0.85 (1) Å, and with *U*_iso_(H) = 1.5 *U*_eq_(O).

### Crystal data

**Table:**

[Co(C_7_H_4_FO_2_)_2_(C_12_H_8_N_2_)(H_2_O)]	*Z* = 2
*M**_r_* = 535.35	*F*(000) = 546
Triclinic, *P*1	*D*_x_ = 1.569 Mg m^−^^3^
Hall symbol: -P 1	Mo *K*α radiation, λ = 0.71073 Å
*a* = 8.6517 (7) Å	Cell parameters from 2450 reflections
*b* = 12.1233 (10) Å	θ = 2.6–26.0°
*c* = 12.6752 (10) Å	µ = 0.82 mm^−^^1^
α = 64.045 (1)°	*T* = 298 K
β = 88.879 (1)°	Block, purple
γ = 72.892 (1)°	0.30 × 0.20 × 0.12 mm
*V* = 1133.22 (16) Å^3^	

### Data collection

**Table:**

Bruker SMART APEXII CCD area-detector diffractometer	3980 independent reflections
Radiation source: fine-focus sealed tube	3119 reflections with *I* > 2σ(*I*)
graphite	*R*_int_ = 0.032
φ and ω scans	θ_max_ = 25.0°, θ_min_ = 1.8°
Absorption correction: multi-scan (*SADABS*; Bruker, 2005)	*h* = −9→10
*T*_min_ = 0.792, *T*_max_ = 0.908	*k* = −14→10
4724 measured reflections	*l* = −14→15

### Refinement

**Table:**

Refinement on *F*^2^	Primary atom site location: structure-invariant direct methods
Least-squares matrix: full	Secondary atom site location: difference Fourier map
*R*[*F*^2^ > 2σ(*F*^2^)] = 0.038	Hydrogen site location: inferred from neighbouring sites
*wR*(*F*^2^) = 0.105	H atoms treated by a mixture of independent and constrained refinement
*S* = 1.03	*w* = 1/[σ^2^(*F*_o_^2^) + (0.055*P*)^2^ + 0.0202*P*] where *P* = (*F*_o_^2^ + 2*F*_c_^2^)/3
3980 reflections	(Δ/σ)_max_ < 0.001
333 parameters	Δρ_max_ = 0.73 e Å^−^^3^
0 restraints	Δρ_min_ = −0.39 e Å^−^^3^

### Special details

**Table:**

Geometry. All e.s.d.'s (except the e.s.d. in the dihedral angle between two l.s. planes) are estimated using the full covariance matrix. The cell e.s.d.'s are taken into account individually in the estimation of e.s.d.'s in distances, angles and torsion angles; correlations between e.s.d.'s in cell parameters are only used when they are defined by crystal symmetry. An approximate (isotropic) treatment of cell e.s.d.'s is used for estimating e.s.d.'s involving l.s. planes.
Refinement. Refinement of *F*^2^ against ALL reflections. The weighted *R*-factor *wR* and goodness of fit *S* are based on *F*^2^, conventional *R*-factors *R* are based on *F*, with *F* set to zero for negative *F*^2^. The threshold expression of *F*^2^ > σ(*F*^2^) is used only for calculating *R*-factors(gt) *etc*. and is not relevant to the choice of reflections for refinement. *R*-factors based on *F*^2^ are statistically about twice as large as those based on *F*, and *R*- factors based on ALL data will be even larger.

### Fractional atomic coordinates and isotropic or equivalent isotropic displacement parameters (Å^2^)

**Table:**

	*x*	*y*	*z*	*U*_iso_*/*U*_eq_	
Co1	0.51799 (4)	0.24471 (3)	0.12214 (3)	0.03633 (15)	
F2	1.0969 (3)	−0.26008 (18)	0.56566 (17)	0.0808 (7)	
F1	0.1529 (3)	0.3143 (2)	0.60217 (19)	0.0947 (8)	
O5	0.6418 (3)	0.32022 (19)	−0.02002 (18)	0.0433 (5)	
O3	0.7374 (2)	0.12837 (18)	0.22283 (17)	0.0447 (5)	
O1	0.4668 (2)	0.41857 (18)	0.16171 (17)	0.0471 (5)	
O2	0.4068 (2)	0.24361 (19)	0.27285 (18)	0.0495 (5)	
O4	0.9083 (2)	0.2289 (2)	0.12131 (18)	0.0584 (6)	
N2	0.4584 (3)	0.0844 (2)	0.13322 (19)	0.0356 (5)	
N1	0.3025 (3)	0.3333 (2)	−0.0037 (2)	0.0407 (6)	
C8	0.8765 (3)	0.1430 (3)	0.2093 (2)	0.0376 (6)	
C25	0.3329 (3)	0.1120 (3)	0.0540 (2)	0.0343 (6)	
C1	0.4124 (3)	0.3528 (3)	0.2530 (2)	0.0380 (7)	
C2	0.3542 (3)	0.4031 (3)	0.3406 (2)	0.0381 (7)	
C9	1.0117 (3)	0.0474 (3)	0.3071 (2)	0.0364 (6)	
C4	0.2237 (4)	0.3823 (3)	0.5107 (3)	0.0573 (8)	
C21	0.2827 (3)	0.0175 (3)	0.0433 (3)	0.0414 (7)	
C26	0.2505 (3)	0.2470 (3)	−0.0217 (2)	0.0354 (6)	
C24	0.5326 (4)	−0.0398 (3)	0.2070 (3)	0.0442 (7)	
H24	0.6190	−0.0609	0.2621	0.053*	
C10	0.9929 (4)	−0.0668 (3)	0.3921 (3)	0.0443 (7)	
H10	0.8981	−0.0870	0.3881	0.053*	
C18	0.1238 (3)	0.2816 (3)	−0.1082 (3)	0.0435 (7)	
C3	0.2792 (3)	0.3340 (3)	0.4326 (3)	0.0455 (7)	
H3	0.2668	0.2570	0.4411	0.055*	
C15	0.2255 (4)	0.4583 (3)	−0.0715 (3)	0.0526 (8)	
H15	0.2583	0.5190	−0.0598	0.063*	
C20	0.1565 (4)	0.0568 (3)	−0.0480 (3)	0.0523 (8)	
H20	0.1251	−0.0058	−0.0575	0.063*	
C22	0.3644 (4)	−0.1114 (3)	0.1232 (3)	0.0519 (8)	
H22	0.3348	−0.1778	0.1201	0.062*	
C14	1.1547 (3)	0.0739 (3)	0.3155 (3)	0.0468 (7)	
H14	1.1689	0.1496	0.2581	0.056*	
C11	1.1172 (4)	−0.1490 (3)	0.4818 (3)	0.0524 (8)	
C7	0.3732 (4)	0.5164 (3)	0.3303 (3)	0.0509 (8)	
H7	0.4254	0.5616	0.2692	0.061*	
C13	1.2761 (4)	−0.0106 (3)	0.4081 (3)	0.0590 (9)	
H13	1.3712	0.0086	0.4134	0.071*	
C17	0.0479 (4)	0.4149 (3)	−0.1789 (3)	0.0569 (9)	
H17	−0.0359	0.4434	−0.2387	0.068*	
C23	0.4859 (4)	−0.1390 (3)	0.2047 (3)	0.0520 (8)	
H23	0.5383	−0.2244	0.2593	0.062*	
C19	0.0811 (4)	0.1834 (3)	−0.1213 (3)	0.0536 (9)	
H19	0.0000	0.2064	−0.1812	0.064*	
C5	0.2395 (4)	0.4944 (3)	0.5024 (3)	0.0651 (10)	
H5A	0.2002	0.5240	0.5573	0.078*	
C6	0.3151 (5)	0.5636 (4)	0.4105 (3)	0.0647 (9)	
H6	0.3268	0.6405	0.4027	0.078*	
C16	0.0966 (4)	0.5018 (3)	−0.1600 (3)	0.0644 (10)	
H16	0.0449	0.5900	−0.2056	0.077*	
C12	1.2573 (4)	−0.1229 (3)	0.4924 (3)	0.0601 (9)	
H12	1.3384	−0.1802	0.5555	0.072*	
H1	0.7360 (17)	0.299 (3)	0.016 (2)	0.071 (12)*	
H2	0.613 (3)	0.4010 (6)	−0.066 (2)	0.067 (11)*	

### Atomic displacement parameters (Å^2^)

**Table:**

	*U*^11^	*U*^22^	*U*^33^	*U*^12^	*U*^13^	*U*^23^
Co1	0.0391 (2)	0.0317 (2)	0.0372 (2)	−0.01239 (17)	0.00096 (17)	−0.01383 (18)
F2	0.1046 (17)	0.0410 (11)	0.0648 (13)	−0.0093 (11)	−0.0002 (12)	−0.0042 (10)
F1	0.1097 (19)	0.1022 (18)	0.0730 (14)	−0.0412 (15)	0.0432 (13)	−0.0369 (13)
O5	0.0520 (14)	0.0336 (12)	0.0385 (12)	−0.0135 (10)	0.0016 (10)	−0.0112 (10)
O3	0.0381 (11)	0.0401 (11)	0.0451 (12)	−0.0147 (9)	−0.0004 (9)	−0.0082 (9)
O1	0.0589 (13)	0.0363 (10)	0.0421 (12)	−0.0120 (10)	0.0096 (10)	−0.0162 (9)
O2	0.0576 (13)	0.0451 (12)	0.0556 (13)	−0.0235 (11)	0.0142 (10)	−0.0269 (11)
O4	0.0484 (13)	0.0549 (14)	0.0503 (13)	−0.0234 (11)	0.0023 (10)	−0.0004 (11)
N2	0.0351 (13)	0.0318 (12)	0.0375 (13)	−0.0105 (10)	0.0061 (10)	−0.0138 (11)
N1	0.0438 (14)	0.0316 (13)	0.0421 (14)	−0.0111 (11)	0.0003 (11)	−0.0131 (11)
C8	0.0397 (17)	0.0350 (15)	0.0408 (16)	−0.0129 (13)	0.0036 (13)	−0.0188 (14)
C25	0.0331 (15)	0.0353 (15)	0.0389 (15)	−0.0129 (12)	0.0101 (12)	−0.0196 (13)
C1	0.0315 (15)	0.0375 (16)	0.0377 (16)	−0.0058 (13)	−0.0031 (13)	−0.0137 (13)
C2	0.0343 (15)	0.0349 (15)	0.0370 (16)	−0.0031 (13)	−0.0038 (12)	−0.0137 (13)
C9	0.0354 (15)	0.0351 (15)	0.0377 (16)	−0.0078 (13)	0.0048 (12)	−0.0177 (13)
C4	0.056 (2)	0.059 (2)	0.0445 (18)	−0.0064 (16)	0.0076 (14)	−0.0200 (16)
C21	0.0423 (17)	0.0457 (17)	0.0499 (18)	−0.0216 (14)	0.0171 (14)	−0.0290 (15)
C26	0.0329 (15)	0.0396 (15)	0.0377 (15)	−0.0136 (13)	0.0068 (12)	−0.0197 (13)
C24	0.0495 (18)	0.0344 (16)	0.0424 (17)	−0.0117 (14)	0.0068 (14)	−0.0130 (14)
C10	0.0444 (17)	0.0380 (17)	0.0479 (18)	−0.0106 (14)	0.0030 (14)	−0.0186 (14)
C18	0.0347 (16)	0.0515 (19)	0.0453 (17)	−0.0140 (14)	0.0047 (13)	−0.0225 (15)
C3	0.0464 (18)	0.0393 (16)	0.0444 (17)	−0.0081 (14)	0.0039 (14)	−0.0168 (14)
C15	0.056 (2)	0.0353 (17)	0.058 (2)	−0.0092 (15)	−0.0075 (16)	−0.0157 (15)
C20	0.0493 (19)	0.067 (2)	0.067 (2)	−0.0310 (18)	0.0162 (17)	−0.0461 (19)
C22	0.062 (2)	0.0423 (19)	0.068 (2)	−0.0268 (17)	0.0216 (18)	−0.0329 (17)
C14	0.0410 (17)	0.0512 (19)	0.0459 (18)	−0.0136 (15)	0.0075 (14)	−0.0205 (15)
C11	0.065 (2)	0.0330 (17)	0.0421 (18)	−0.0021 (16)	0.0036 (16)	−0.0106 (15)
C7	0.060 (2)	0.0481 (18)	0.0477 (18)	−0.0203 (17)	0.0085 (16)	−0.0227 (16)
C13	0.0378 (18)	0.075 (3)	0.056 (2)	−0.0111 (17)	0.0021 (16)	−0.027 (2)
C17	0.0423 (18)	0.062 (2)	0.058 (2)	−0.0108 (17)	−0.0092 (16)	−0.0229 (18)
C23	0.062 (2)	0.0299 (16)	0.057 (2)	−0.0140 (15)	0.0136 (17)	−0.0144 (15)
C19	0.0384 (18)	0.080 (3)	0.057 (2)	−0.0253 (18)	0.0046 (15)	−0.040 (2)
C5	0.072 (2)	0.067 (2)	0.060 (2)	−0.009 (2)	0.0108 (19)	−0.040 (2)
C6	0.079 (2)	0.059 (2)	0.065 (2)	−0.021 (2)	0.011 (2)	−0.0368 (19)
C16	0.060 (2)	0.047 (2)	0.061 (2)	−0.0019 (18)	−0.0139 (18)	−0.0116 (18)
C12	0.048 (2)	0.062 (2)	0.050 (2)	0.0102 (18)	−0.0089 (16)	−0.0242 (18)

### Geometric parameters (Å, °)

**Table:**

Co1—O3	2.0412 (19)	C26—C18	1.397 (4)
Co1—O5	2.071 (2)	C24—C23	1.388 (4)
Co1—N2	2.101 (2)	C24—H24	0.9300
Co1—O2	2.118 (2)	C10—C11	1.367 (4)
Co1—N1	2.147 (2)	C10—H10	0.9300
Co1—O1	2.2930 (19)	C18—C17	1.406 (4)
F2—C11	1.361 (4)	C18—C19	1.420 (4)
F1—C4	1.354 (4)	C3—H3	0.9300
O5—H1	0.8500 (11)	C15—C16	1.403 (4)
O5—H2	0.8499 (11)	C15—H15	0.9300
O3—C8	1.265 (3)	C20—C19	1.353 (4)
O1—C1	1.261 (3)	C20—H20	0.9300
O2—C1	1.250 (3)	C22—C23	1.348 (4)
O4—C8	1.243 (3)	C22—H22	0.9300
N2—C24	1.333 (3)	C14—C13	1.375 (4)
N2—C25	1.354 (3)	C14—H14	0.9300
N1—C15	1.330 (3)	C11—C12	1.362 (5)
N1—C26	1.356 (3)	C7—C6	1.390 (4)
C8—C9	1.500 (4)	C7—H7	0.9300
C25—C21	1.397 (4)	C13—C12	1.367 (5)
C25—C26	1.440 (4)	C13—H13	0.9300
C1—C2	1.498 (4)	C17—C16	1.352 (5)
C2—C7	1.382 (4)	C17—H17	0.9300
C2—C3	1.385 (4)	C23—H23	0.9300
C9—C14	1.384 (4)	C19—H19	0.9300
C9—C10	1.387 (4)	C5—C6	1.387 (5)
C4—C5	1.364 (5)	C5—H5A	0.9300
C4—C3	1.370 (4)	C6—H6	0.9300
C21—C22	1.400 (4)	C16—H16	0.9300
C21—C20	1.421 (4)	C12—H12	0.9300
			
O3—Co1—O5	88.75 (8)	C23—C24—H24	118.7
O3—Co1—N2	91.47 (8)	C11—C10—C9	118.3 (3)
O5—Co1—N2	113.37 (8)	C11—C10—H10	120.9
O3—Co1—O2	91.55 (8)	C9—C10—H10	120.9
O5—Co1—O2	152.67 (8)	C26—C18—C17	116.3 (3)
N2—Co1—O2	93.94 (8)	C26—C18—C19	119.6 (3)
O3—Co1—N1	165.19 (8)	C17—C18—C19	124.1 (3)
O5—Co1—N1	86.87 (9)	C4—C3—C2	118.1 (3)
N2—Co1—N1	77.36 (8)	C4—C3—H3	121.0
O2—Co1—N1	98.81 (8)	C2—C3—H3	121.0
O3—Co1—O1	101.55 (8)	N1—C15—C16	122.3 (3)
O5—Co1—O1	94.09 (8)	N1—C15—H15	118.9
N2—Co1—O1	149.92 (8)	C16—C15—H15	118.9
O2—Co1—O1	59.10 (7)	C19—C20—C21	121.3 (3)
N1—Co1—O1	92.87 (8)	C19—C20—H20	119.4
Co1—O5—H1	101 (2)	C21—C20—H20	119.4
Co1—O5—H2	121 (2)	C23—C22—C21	119.9 (3)
H1—O5—H2	107.6 (19)	C23—C22—H22	120.1
C8—O3—Co1	129.55 (18)	C21—C22—H22	120.1
C1—O1—Co1	86.01 (16)	C13—C14—C9	120.6 (3)
C1—O2—Co1	94.26 (17)	C13—C14—H14	119.7
C24—N2—C25	117.4 (2)	C9—C14—H14	119.7
C24—N2—Co1	127.2 (2)	F2—C11—C12	119.1 (3)
C25—N2—Co1	115.39 (17)	F2—C11—C10	117.9 (3)
C15—N1—C26	117.5 (2)	C12—C11—C10	123.0 (3)
C15—N1—Co1	128.8 (2)	C2—C7—C6	120.6 (3)
C26—N1—Co1	113.42 (17)	C2—C7—H7	119.7
O4—C8—O3	124.8 (3)	C6—C7—H7	119.7
O4—C8—C9	118.6 (2)	C12—C13—C14	120.2 (3)
O3—C8—C9	116.6 (2)	C12—C13—H13	119.9
N2—C25—C21	123.4 (3)	C14—C13—H13	119.9
N2—C25—C26	116.4 (2)	C16—C17—C18	120.1 (3)
C21—C25—C26	120.1 (2)	C16—C17—H17	119.9
O2—C1—O1	120.6 (3)	C18—C17—H17	119.9
O2—C1—C2	118.6 (3)	C22—C23—C24	119.9 (3)
O1—C1—C2	120.9 (3)	C22—C23—H23	120.1
C7—C2—C3	120.0 (3)	C24—C23—H23	120.1
C7—C2—C1	121.2 (3)	C20—C19—C18	120.9 (3)
C3—C2—C1	118.7 (3)	C20—C19—H19	119.5
C14—C9—C10	119.3 (3)	C18—C19—H19	119.5
C14—C9—C8	120.3 (2)	C4—C5—C6	118.7 (3)
C10—C9—C8	120.4 (2)	C4—C5—H5A	120.6
F1—C4—C5	118.2 (3)	C6—C5—H5A	120.6
F1—C4—C3	118.5 (3)	C5—C6—C7	119.2 (3)
C5—C4—C3	123.3 (3)	C5—C6—H6	120.4
C25—C21—C22	116.8 (3)	C7—C6—H6	120.4
C25—C21—C20	118.9 (3)	C17—C16—C15	119.7 (3)
C22—C21—C20	124.3 (3)	C17—C16—H16	120.2
N1—C26—C18	124.1 (3)	C15—C16—H16	120.2
N1—C26—C25	116.8 (2)	C11—C12—C13	118.6 (3)
C18—C26—C25	119.1 (3)	C11—C12—H12	120.7
N2—C24—C23	122.6 (3)	C13—C12—H12	120.7
N2—C24—H24	118.7		
			
O5—Co1—O3—C8	23.8 (2)	N2—C25—C21—C22	2.8 (4)
N2—Co1—O3—C8	137.1 (2)	C26—C25—C21—C22	−177.4 (3)
O2—Co1—O3—C8	−128.9 (2)	N2—C25—C21—C20	−175.9 (3)
N1—Co1—O3—C8	96.5 (4)	C26—C25—C21—C20	3.9 (4)
O1—Co1—O3—C8	−70.2 (2)	C15—N1—C26—C18	1.3 (4)
O3—Co1—O1—C1	−83.30 (16)	Co1—N1—C26—C18	−172.9 (2)
O5—Co1—O1—C1	−172.86 (16)	C15—N1—C26—C25	−178.8 (3)
N2—Co1—O1—C1	30.6 (2)	Co1—N1—C26—C25	7.0 (3)
O2—Co1—O1—C1	1.53 (15)	N2—C25—C26—N1	−2.2 (4)
N1—Co1—O1—C1	100.08 (16)	C21—C25—C26—N1	177.9 (2)
O3—Co1—O2—C1	101.01 (17)	N2—C25—C26—C18	177.7 (2)
O5—Co1—O2—C1	10.7 (3)	C21—C25—C26—C18	−2.1 (4)
N2—Co1—O2—C1	−167.41 (16)	C25—N2—C24—C23	0.0 (4)
N1—Co1—O2—C1	−89.61 (17)	Co1—N2—C24—C23	−178.2 (2)
O1—Co1—O2—C1	−1.54 (15)	C14—C9—C10—C11	0.4 (4)
O3—Co1—N2—C24	13.8 (2)	C8—C9—C10—C11	−177.7 (2)
O5—Co1—N2—C24	103.0 (2)	N1—C26—C18—C17	−0.3 (4)
O2—Co1—N2—C24	−77.9 (2)	C25—C26—C18—C17	179.7 (3)
N1—Co1—N2—C24	−176.0 (2)	N1—C26—C18—C19	178.7 (3)
O1—Co1—N2—C24	−102.6 (3)	C25—C26—C18—C19	−1.2 (4)
O3—Co1—N2—C25	−164.44 (18)	F1—C4—C3—C2	−178.8 (3)
O5—Co1—N2—C25	−75.2 (2)	C5—C4—C3—C2	−0.3 (5)
O2—Co1—N2—C25	103.90 (18)	C7—C2—C3—C4	0.8 (4)
N1—Co1—N2—C25	5.74 (18)	C1—C2—C3—C4	−178.8 (3)
O1—Co1—N2—C25	79.2 (2)	C26—N1—C15—C16	−0.9 (5)
O3—Co1—N1—C15	−138.4 (3)	Co1—N1—C15—C16	172.2 (2)
O5—Co1—N1—C15	−65.4 (3)	C25—C21—C20—C19	−2.3 (4)
N2—Co1—N1—C15	179.8 (3)	C22—C21—C20—C19	179.1 (3)
O2—Co1—N1—C15	87.7 (3)	C25—C21—C22—C23	−0.5 (4)
O1—Co1—N1—C15	28.6 (3)	C20—C21—C22—C23	178.1 (3)
O3—Co1—N1—C26	35.0 (4)	C10—C9—C14—C13	−1.2 (4)
O5—Co1—N1—C26	107.98 (19)	C8—C9—C14—C13	176.9 (3)
N2—Co1—N1—C26	−6.82 (18)	C9—C10—C11—F2	179.2 (3)
O2—Co1—N1—C26	−98.91 (19)	C9—C10—C11—C12	0.9 (5)
O1—Co1—N1—C26	−158.07 (19)	C3—C2—C7—C6	−1.1 (5)
Co1—O3—C8—O4	−7.9 (4)	C1—C2—C7—C6	178.4 (3)
Co1—O3—C8—C9	173.02 (16)	C9—C14—C13—C12	0.8 (5)
C24—N2—C25—C21	−2.5 (4)	C26—C18—C17—C16	−1.1 (5)
Co1—N2—C25—C21	175.9 (2)	C19—C18—C17—C16	180.0 (3)
C24—N2—C25—C26	177.7 (2)	C21—C22—C23—C24	−1.8 (5)
Co1—N2—C25—C26	−3.9 (3)	N2—C24—C23—C22	2.1 (5)
Co1—O2—C1—O1	2.8 (3)	C21—C20—C19—C18	−1.1 (5)
Co1—O2—C1—C2	−176.7 (2)	C26—C18—C19—C20	2.8 (4)
Co1—O1—C1—O2	−2.6 (2)	C17—C18—C19—C20	−178.2 (3)
Co1—O1—C1—C2	176.9 (2)	F1—C4—C5—C6	178.6 (3)
O2—C1—C2—C7	173.0 (3)	C3—C4—C5—C6	0.1 (5)
O1—C1—C2—C7	−6.5 (4)	C4—C5—C6—C7	−0.4 (5)
O2—C1—C2—C3	−7.4 (4)	C2—C7—C6—C5	0.9 (5)
O1—C1—C2—C3	173.1 (2)	C18—C17—C16—C15	1.4 (5)
O4—C8—C9—C14	17.7 (4)	N1—C15—C16—C17	−0.4 (5)
O3—C8—C9—C14	−163.2 (3)	F2—C11—C12—C13	−179.6 (3)
O4—C8—C9—C10	−164.2 (3)	C10—C11—C12—C13	−1.3 (5)
O3—C8—C9—C10	14.9 (4)	C14—C13—C12—C11	0.5 (5)

### Hydrogen-bond geometry (Å, °)

**Table:**

*D*—H···*A*	*D*—H	H···*A*	*D*···*A*	*D*—H···*A*
O5—H1···O4	0.85 (1)	1.76 (1)	2.584 (3)	165 (3)
O5—H2···O1^i^	0.85 (1)	1.89 (1)	2.734 (3)	176 (3)
C22—H22···O5^ii^	0.93	2.52	3.332 (4)	147.
C5—H5A···F2^iii^	0.93	2.55	3.303 (4)	138.

Symmetry codes: (i) −*x*+1, −*y*+1, −*z*; (ii) −*x*+1, −*y*, −*z*; (iii) *x*−1, *y*+1, *z*.
